# Telepractice in School-Age Children Who Stutter: A Controlled Before and After Study to Evaluate the Efficacy Of MIDA-SP

**DOI:** 10.5195/ijt.2021.6380

**Published:** 2021-06-22

**Authors:** Donatella Tomaiuoli, Francesca Del Gado, Sara Marchetti, Lisa Scordino, Diletta Vedovelli

**Affiliations:** 1 CRC Center of Research and Cure of Rome, Rome, Italy; 2 Sapienza University of Rome, Rome, Italy; 3 University of Rome Tor Vergata, Rome, Italy

**Keywords:** Fluency, MIDA-Stuttering Program, Stuttering, Telehealth, Telepractice

## Abstract

The COVID-19 pandemic necessitated a general reorganization of rehabilitation services in Italy. The lockdown in Italy led to the use of telepractice for the delivery of speech therapy, including stuttering. The aim of the present work was to evaluate the effectiveness of the Multidimensional, Integrated, Differentiated, Art-Mediated Stuttering Program (MIDA-SP; Tomaiuoli et al., 2012), delivered online for school-age children who stutter. A non-randomized controlled pre- and post-treatment study included an experimental group (11 children) receiving a telepractice adaptation of MIDA-SP and a historical control group (11 children) receiving in-person MIDA-SP. Both groups had been assessed with the Stuttering Severity Instrument – Fourth Edition (SSI-4) and Overall Assessment of the Speaker's Experience of Stuttering (OASES-S) pre- and post-treatment. No statistically significant differences were found between the two modes of delivery. These findings suggest that MIDA-SP treatment delivered via telepractice is effective for school-age children who stutter.

In February 2020, cases of acute pneumonia caused by a new Coronavirus, named Severe Acute Respiratory Syndrome Coronavirus 2 (SARS-COV-2), were reported in Italy. This virus can cause severe respiratory problems, especially in the elderly and immunocompromised individuals. Given the global spread of the infection, in March 2020 the World Health Organization declared that the syndrome caused by Sars-Cov-2 was a pandemic and named it COVID-19 (Ludwig & Zarbock, 2020). From March through May 2020, the Italian Government banned any type of travel throughout the national territory if not for actual and demonstrated need. A general reorganization of rehabilitation services was necessary to face this pandemic. Telepractice was introduced to ensure continuity of treatment and uninterrupted service delivery.

Telepractice consists of the delivery of clinical and rehabilitation services through modern communication technologies such as phone, email, chat, mobile apps, and live-stream videoconferencing platforms (American Speech-Language-Hearing Association, 2019).

This relatively new mode of delivery brought several advantages. Telepractice eliminates geographical barriers, addresses some of the difficulties in finding experts, minimizes limitations caused by mobility, leads to a reduction in costs, and allows for elimination of travel time and time spent in the waiting room (Coufal et al., 2017; Covert et al., 2018; Weidner & Lowman, 2020). Thus, telepractice could be an effective treatment delivery method in circumstances for which travel limitations would make in-person treatment difficult. Telepractice can provide a direct connection between a therapist and a patient via a telematic mode.

Covert et al. (2018) examined the effect of telepractice on missed appointment rates using a randomized control trial (RCT). In this study, 36 participants (34 males, 2 females) were divided into two groups: the experimental group, that received speech therapy via telepractice (TR), and the control group, that received in-person treatment (IP). All participants received as treatment LSVT©, an intensive rehabilitation technique designed specifically for Parkinson's disease. Results show that the group that received treatment by telepractice had completed more therapy sessions than the control group (TR:13.27 sessions; IP:10.5 sessions). These findings suggest that telepractice service delivery can provide greater access to services. Missed appointments cause financial losses for clinics and affect the continuity of the therapeutic rehabilitation process for patients. Numerous factors can lead to the cancellation of an appointment, with most linked to logistical challenges (Tindall et al., 2008). These include distance from the place of therapy, scheduling considerations, meeting the needs of other children in the family, and cost of travel.

Many studies have examined the effectiveness of treatment for stuttering delivered by telepractice (Bridgman et al., 2016; Cangi & Toğram, 2020; Carey et al, 2010, 2014; Eslami Jahromi et al., 2020; Kully, 2000; Lowe et al., 2013; Valentine, 2015). Specifically, Carey et al. (2014) and Eslami Jahromi et al. (2020) investigated the effectiveness of the Camperdown Program delivered by telepractice. The Camperdown Program treatment is based on the principle of speech restructuring (O’Brian et al, 2018). This program involves both individual and group therapy. There are two goals of the program: (1) the elimination or significant reduction of disfluencies, and (2) helping the client develop strategies that will allow them to independently manage stuttering and any related anxiety. The intervention program is divided into four phases. As the program progresses, meetings with the speech-language pathologist will be less and less frequent. In both of the aforementioned studies, the results showed no significant differences between the Camperdown Program delivered in-person and delivered electronically.

Bridgman et al. (2016) aimed to investigate the effectiveness of the Lidcombe Program delivered by telepractice. The Lidcombe Program is an integrated intervention program consisting of behavioral treatment designed for preschool children (Onslow et al., 2003). The program does not reference any causal theory of stuttering (Onslow & Millard, 2012) and is based on setting up specific ways to respond to moments of disfluent speech through parent training. Bridgman et al. (2016) carried out an RCT comparing the outcomes of the treatment delivered by telepractice (experimental group) with those of the in-person treatment (control group), in a sample of 49 pre-school children. The data collected showed comparable outcomes between the two modes of delivery.

Cangi and Toğram (2020) investigated the effectiveness of stuttering treatment delivered by telepractice on a sample of 20 adults who stutter, comparing it with the same treatment delivered in-person. This was a non-inferiority-controlled trial. The data showed no statistically significant difference between the results obtained for the two groups. These findings suggest that telepractice is a suitable method for adults who stutter, especially for those who have difficulties in accessing in-person services.

According to the results obtained in the studies mentioned above, telepractice can be considered an effective method in rehabilitation for individuals who stutter. In addition, telepractice does not lead to a lowering of the quality standard compared to the same treatment provided in-person.

Over the years, studies on the effectiveness of telepractice have mainly focused on pre-school, adolescent, or adult age groups. There are fewer studies investigating the effectiveness of the treatment in school-age children. This paucity of research is important because school-age children go through many changes. It is during this period that the transition from pre-school to primary school takes place, wherein children must interface with new teachers, new classmates, and will experience new contexts and activities (e.g., answering questions, reading aloud, first presentations). In addition, from the age of 6 years on, opportunities for socializing increase. Children begin to experience various types of leisure activities, such as sports, theatre, or music lessons; these will lay the foundations for their future passions and nurture their social relationships. The school years bring new situations and challenges, including the increased demands of the environment and the need to deal with new teachers and peers. Therefore, it not surprising that children who stutter may encounter some difficulties as they navigate the challenges presented by attending school.

## MIDA-STUTTERING PROGRAM

The MIDA-Stuttering Program (Tomaiuoli et al., 2012) is a Multidimensional, Integrated, Differentiated and Art-mediated rehabilitation program for people who stutter. The MIDA-SP initially provides a multidimensional assessment of overt and covert aspects of stuttering in the individual who stutters, to derive a severity profile. According to Sheehan's metaphor (1970), stuttering is seen as an iceberg: the tip of the iceberg would represent the overt aspects, such as disfluencies and the related visible behaviors. The deeper and more hidden part of the iceberg is the covert aspects which consist of the feelings, thoughts and attitude related to the disorder.

Based on the profile, macro and micro aims are next outlined. The MIDA-SP incorporates principles and strategies from both the stuttering modification and fluency shaping approaches. The program includes the acquisition of verbal facilitation techniques to shape the fluency, and the practice of pseudo-stuttering to reduce fear, anxiety, and other negative emotions and thoughts about stuttering (Davidow et al., 2019). The therapy sessions are integrated with Transfer Activities and Mediated Art Training, to allow the automation and generalization of verbal facilitation techniques. The program incorporates children's participation in an activity to be chosen by the rehabilitation team: theatrical path with final performance, dubbing, and radio. The therapeutic path thus structures aims to increase the self-esteem and sense of self-efficacy of clients, as well as to promote the use of verbal facilitation techniques, the acquisition of which, however, does not represent the true focus of the program.

## PURPOSE

This trial was designed in the face of the reorganization mentioned above. It aimed to investigate the effectiveness of the intervention program for developmental stuttering MIDA-SP (Multidimensional, Integrated, Differentiated, Art-Mediated Stuttering Program) (Tomaiuoli et al., 2012) delivered by telepractice in a school-age group, and to compare the results to those obtained through the same treatment delivered in-person in a historical control group.

While there are numerous efficacy studies from other countries (e.g., Australia, Turkey), the effectiveness of telepractice in the field of stuttering has not been previously investigated in Italy. Furthermore, the studies identified internationally have concentrated only minimally on the school-age group (7–13 years).

The three-fold aims of this study were to:

1. compare the effectiveness of the telepractice delivered MIDA-Stuttering Program (Tomaiuoli et al., 2012) and in-person therapy delivery in two school-age groups,
2. report upon a first experience of using telepractice in Italy in the treatment of stuttering,
3. compare missed appointment rates between telepractice and in-person therapy conditions to determine whether telepractice could improve treatment continuity.

## DESIGN AND METHODS

### DESIGN

The present study evaluated the effectiveness of the MIDA-SP program for the treatment of developmental stuttering (Tomaiuoli et al., 2012) delivered by telepractice, and compared it with the same treatment delivered in-person in a historical control group in school age children. The design is a non-randomized controlled pre- and post-treatment trial. This study was approved by an internal scientific committee. The therapies and projects provided at this Center were approved by the Italian National Health System.

### PARTICIPANTS AND SETTINGS

This non-randomized controlled pre- and post-treatment trial included two groups: an experimental group (11 children who stutter; 10 males and 1 female; mean age of 9,30 years) receiving telehealth adaptation of MIDA-SP (i.e., Telepractice Group); and an historical control group (11 children who stutter; 10 males and 1 female; mean age of 9,48 years) receiving MIDA-SP at Center of Research and Cure (CRC) based in Rome (i.e., In-Clinic Group). Initially, 13 patients were recruited into the historical control group, but two were excluded to make the two groups more comparable for age. Participant selection criteria required that (a) participants stuttered; (b) participants were between 7 and 12 years at the beginning of the study; (c) participants were reported by parents to have been stuttering for at least six months at the beginning of the study; (d) participants had not received treatment for stuttering for at least two months prior to treatment; (e) informed consent to participate in the research was obtained from the child's parents. Exclusion criteria were comorbidity with other neurodevelopmental disorders.

**Table 1:** Baseline Characteristics of Patients in Telepractice

NAME	GENDER	AGE (years)	FAMILY HISTORY OF STUTTERING	SSI-4 PRE	OASES PRE
T1	M	8,7	N	18	2
T2	M	10,4	N	40	1,5
T3	M	7,7	Y	20	1,6
T4	M	7,1	N	27	1,7
T5	M	7,8	Y	32	1,6
T6	M	7,4	N	20	1,4
T7	M	8	N	30	1,6
T8	F	11,7	N	19	2,6
T9	M	10,9	N	26	1,8
T10	M	10,7	N	30	2,9
T11	M	12	N	23	2,82
**MEAN**		9,31		25,91	1,96
**STANDARD DEVIATION**		1,85		6,77	0,55

**Table 2:** Baseline Characteristics of Patients in Clinic Therapy

NAME	GENDER	AGE (years)	FAMILY HISTORY OF STUTTERING	SSI-4 PRE	OASES PRE
IC1	M	8,7	Y	36	2,98
IC2	M	9,7	N	41	2,2
IC3	M	7	N	24	2,42
IC4	M	9,8	Y	30	1,7
IC5	M	9,8	Y	34	1,8
IC6	M	10,5	Y	24	1,8
IC7	M	7,8	Y	24	2
IC8	F	7,6	Y	28	2,3
IC9	M	10,9	N	19	3,2
IC10	M	10,6	N	16	2,5
IC11	M	12	N	14	1,8
**MEAN**		9,49		26,36	2,25
**STANDARD DEVIATION**		1,55		8,44	0,50

### INTERVENTIONS

A control group was recruited in September 2019 and received MIDA-SP for 60 hours. An experimental group was recruited in April 2020 and received telehealth adaptation of MIDA-SP for 60 hours.

Due to the COVID-19 pandemic, the program was readjusted in accordance with preventive measures against the virus. While it was not possible to carry out the Art-mediated training, the Transfer activities were enhanced. These activities allowed clients to experiment and generalize the fluency-enhancing techniques learned during the therapy sessions and to reduce avoidance behaviors in real-life speaking contexts. These activities were proposed according to an increasing level of difficulty and considered the communication needs of the patient. Therapy activities were: (1) use of the telephone, (2) conversation, (3) public speaking, (4) simulation of daily living, and (5) role playing of situations. In the treatment delivered by telepractice all activities were carried out using software that enabled video and voice one-to-one calls, group calls, and file sharing.

The program provided: a first phase characterized by individual therapy sessions with two sessions per week lasting an hour and a half each, and a second phase characterized by the introduction of one session per week of group therapy lasting about an hour and a half. The telepractice delivery method through videoconferencing had mainly the same aims and structure of in-person therapy: duration of treatment of six months; two sessions per week; ninety minutes per session; individual and group therapy; use of Transfer Activities; and a maximum of four children in group therapy.

### MEASUREMENT TOOLS

Primary outcomes were the total scores on the Stuttering Severity Instrument - Fourth Edition (SSI-4; Riley, 2009) and Overall Assessment of the Speaker's Experience of Stuttering (OASES-S; Yaruss & Quesal, 2006) obtained at the end of the treatment. A secondary outcome was the number of absences from therapy.

Assessment procedures were conducted by the clinician, immediately pre-treatment and at the end of the program. The evaluation of the in-person group was carried out within the clinic, while the telepractice group was evaluated online.

#### STUTTERING SEVERITY INSTRUMENT (SSI-4)

The SSI-4 procedure (Riley, 2009) was used in the present study. The SSI-4 provides information about stuttering severity in terms of frequency, duration of stuttering events and physical concomitants. Frequency score is based on the percentage of stuttered syllables (%SS) in spontaneous speech and reading tasks and converted to scale score of 2–18 point. The duration score weights the average of the three longest disfluencies. Ratings of physical concomitants are based on tester observations during the speech sample collection. For each of four different areas (i.e., distracting sounds, facial grimaces, head movement, movements of the extremities), a score from 0 to 5 is assigned. A total score is obtained adding together the scores followed by conversion in a percentile rank or in a severity equivalent judgment ranging from very mild to very severe.

#### OVERALL ASSESSMENT OF THE SPEAKER’S EXPERIENCE OF STUTTERING (OASES)

Since the OASES process of validation in Italy is not yet concluded, we used a translated author-reviewed version of OASES for school-aged children, OASES-S. The scale consists of 60 items, divided into four sections: (1) general information about the speaker's perceptions of stuttering, (2) the speaker's reactions to stuttering, (3) difficulties with communication in daily situations and (4) overall impact on quality of life. Each question is scored on a 5-point Likert scale. Responses are totaled into Impact Scores and Impact Ratings (Mild through Severe).

### DATA ANALYSES

The variables considered for this study are gender, age, SSI-4 pre and post values, and OASES pre and post values.

First, the means and the standard deviations of the quantitative variables of the two groups were calculated. Subsequently, parametric and non-parametric tests were performed, due to the low sample size.

To analyze the differences between the SSI-4 pre and post values in each group we used:

– the paired-sample t test: The null hypothesis of the test is that the means in the two variables are equal, against the alternative hypothesis that the means are different.
– the Wilcoxon-Signed Rank Sum test: The null hypothesis of the test is that the medians in the two variables are not significantly different, against the alternative hypothesis that they are. The test statistic is given by the sum of the ranks.

The same procedure was used to analyze whether there were statistically significant differences between the OASES pre and post values in each group.

The t test of independent sample and the Mann Whitney test was used to analyze the differences between the values of each variable (SSI-4 pre, SSI-4 post, OASES pre, OASES post and Missed Appointment Rate) in the two groups. For the t test, the null hypothesis was that the mean of the two subgroups were not significantly different, while the alternative hypothesis was that there was a statistically significant difference. For the Mann Whitney test, the null hypothesis was that the medians of the two subgroups were not significantly different, while the alternative hypothesis is that there was a statistically significant difference. This test statistic is also based on rank sums.

Finally, a post-hoc power analysis on the three outcome variables was done to evaluate if the non-significant results could be attributed to an insufficient sample size. In terms of significance level, p <0.05 was considered statistically significant. Statistical analysis of the data was performed with IBM SPSS Statistics v25 software.

## RESULTS

**Figure 1:**
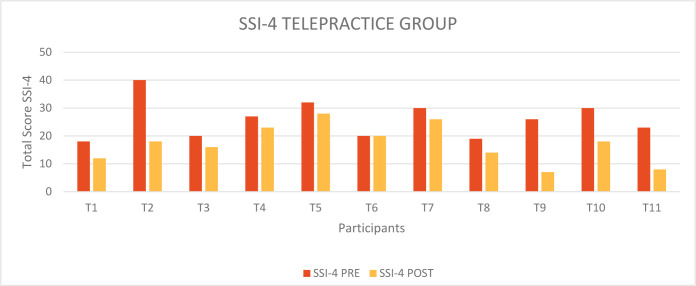
SSI-4 total score pre and post treatment Telepractice Group

The following figures and tables illustrate the results obtained by both groups at the assessments conducted through the administration of the SSI-4 and OASES tests before and after receiving treatment. The data obtained were then compared and statistically analyzed through the application of Student's t-test (Dancey et al., 2012). **Figure 1** shows the total scores obtained by the individual participants of the Telepractice Group on the SSI-4 test before (red columns) and after (orange columns) receiving treatment by telepractice. In almost all individuals there was a decrease in the total severity score; only in one case, T6, there was no change before and after treatment, the child in fact remains at a total score of 20 (Mild). We registered an average pre-treatment score of 25.9 (Moderate), and an average post-treatment score of 17.3 (Mild); the difference between the two scores is therefore equal to 8.6. To verify the effectiveness of the MIDA-SP treatment delivered in telepractice on overt aspects of stuttering, the data were statistically analyzed through the application of a two-tailed Student's T test for paired data and Wilcoxon-Signed Rank Sum test for paired data. We obtained t_10_=3.978; p=00.003 and W_10_=−2.821; p=0.005. Therefore, there was a statistically significant difference between the results obtained before and after treatment, that is, before treatment the values were higher.

**Figure 2:**
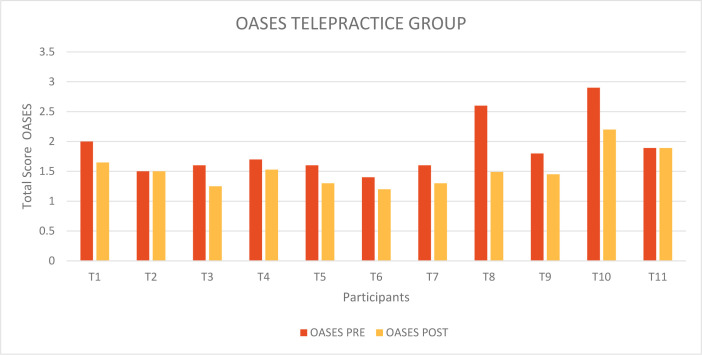
OASES Total Score pre and post treatment Telepractice Group

**Figure 2** shows the total scores obtained by the participants in the Telepractice Group on the OASES questionnaire before (red columns) and after (orange columns) receiving treatment delivered by telepractice.

In almost all individuals, there was a decrease in the total severity score; only in T2 and T11 was there no change in the score, the children scoring at 1.5 (Mild-Moderate) and 1.89 (Mild-Moderate), respectively.

There was a change from a mean pre-treatment score of 1.87 (Mild-Moderate) to a mean post-treatment score of 1.52 (Mild-Moderate); the difference between the two scores is therefore 0.35.

The data were also statistically analyzed through the application of the two-tailed Student's T test for paired data and Wilcoxon-Signed Rank Sum test for paired data, to evaluate the effectiveness of the MIDA-SP treatment delivered through telepractice on improvement of the covert aspects of stuttering. We obtained t_10_=4.224; p=0.002 and W_10_ = −2.812; p=0.005. Therefore, a statistically significant difference was found between the results obtained before and after receiving the treatment.

**Table 3** summarizes the scores obtained by participants in the Telepractice Group before and after receiving treatment on the SSI-4 and OASES tests.

**Table 3:** SSI-4 and OASES's total scores pre and post treatment Telepractice Group

NAME	SSI-4 PRE	SSI-4 POST	OASES PRE	OASES POST
T1	18	12,0	2	1,65
T2	40	18	1,5	1,5
T3	20	16	1,6	1,25
T4	27	23	1,7	1,53
T5	32	28	1,6	1,3
T6	20	20	1,4	1,2
T7	30	26	1,6	1,3
T8	19	14	2,6	1,49
T9	26	7	1,8	1,45
T10	30	18	2,9	2,2
T11	23	8	1,89	1,89

**Figure 3:**
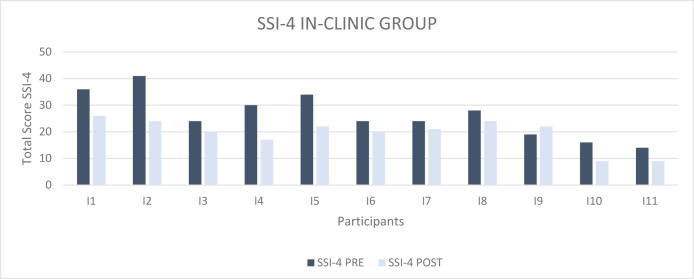
SSI-4 total score pre and post treatment In-Clinic Group

**Figure 3** shows the total scores obtained by individual participants in the In-Clinic group on the SSI-4 test before (dark blue columns) and after (light blue columns) receiving treatment.

All individuals belonging to the group improved their fluency; in fact, there was a decrease in the total severity score for all subjects.

We recorded an average pre-treatment score of 26.4 (Moderate), and an average post-treatment score of 19.5 (Mild); the difference between the two scores is therefore equal to 6.9.

To verify the effectiveness of the MIDA-SP treatment delivered in-person on improving fluency, these data were statistically analyzed through the application of a two-tailed Student's T test for paired data and Wilcoxon-Signed Rank Sum test for paired data. We obtained t_10_=4.071; p=0.002 and W_10_=−2.808; p=0.005. Therefore, there was a statistically significant difference between the results obtained before and after treatment.

**Figure 4:**
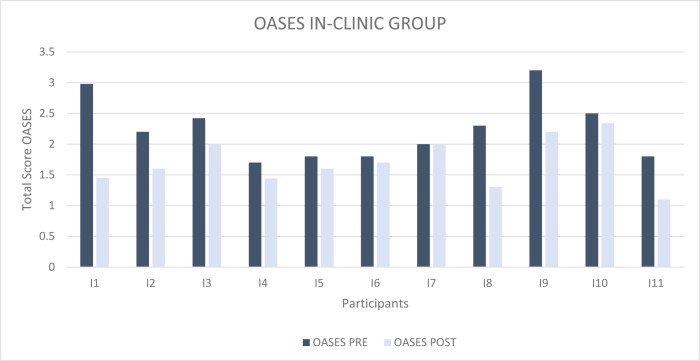
OASES Total Score pre and post treatment for the In-Clinic Group

**Figure 4** shows the total scores obtained by participants in the In-Clinic Group on the OASES questionnaire before (dark blue columns) and after (light blue columns) receiving treatment.

A decrease of the total severity score was observed in all individuals.

We registered a mean pre-treatment score of 2.24 (Mild-Moderate) and a mean post-treatment score of 1.63 (Mild-Moderate); the difference between the two scores was therefore 0,61.

Also in this graph, the data were statistically analyzed through the application of the two-tailed Student's T test for paired data and Wilcoxon-Signed Rank Sum test for paired data, to evaluate the effectiveness of the MIDA-SP treatment delivered in-person on the improvement of covert aspects. We obtained t_10_=3.778; p=0.004 and W_10_==−2.805; p= 0.005. Therefore, a statistically significant difference was found between the results obtained before and after receiving the treatment.

**Table 4** summarizes the scores obtained by individual participants in the In-Clinic group on the SSI-4 and OASES tests before and after treatment.

**Table 4:** SSI-4 and OASES's Total Scores pre and post treatment for the In-Clinic Group

NAME	SSI-4 PRE	SSI-4 POST	OASES PRE	OASES POST
I1	36	26,0	2,98	1,45
I2	41	24	2,2	1,6
I3	24	20	2,42	2
I4	30	17	1,7	1,44
I5	34	22	1,8	1,6
I6	24	20	1,8	1,7
I7	24	21	2	2
I8	28	24	2,3	1,3
I9	19	22	3,2	2,2
I10	16	9	2,5	2,34
I11	14	9	1,8	1,1

**Figure 5:**
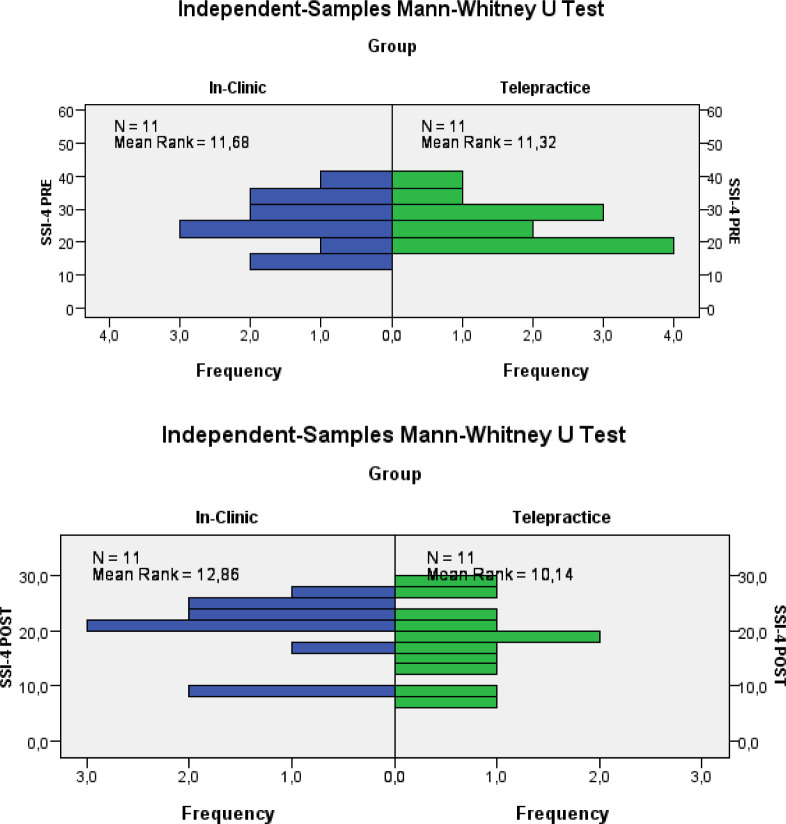
Comparison SSI-4's Total Score

**Figure 5** compares the results obtained at the pre- and post-treatment assessments of the two groups on the SSI-4 test.

With the aim of statistically comparing the results obtained through the two different modes of delivery, a statistical analysis was performed through the application of a two-tailed Student's T test for independent groups and the Mann Whitney test for independent groups. For SSI-4 pre we obtained t_20_=−0.139; p=0.891 and Mann Whitney U= 62.5; n1 = n2 = 11; p= 0.898 and for SSI-4 post we obtained t_20_=−0.815; p=0.425 and Mann Whitney U =75.5; n1 = n2 = 11; p= 0.332. This data indicates that there is no statistically significant difference between the results for the In-Clinic Group and the Telepractice Group on the improvement of overt aspects of stuttering.

We calculated the power of the two tests, which is the probability of correctly rejecting the null hypothesis when it is false. In the first case the power is equal to 3.4%, or the II type error (1-power) is equal to 96.6%, while in the second case it is equal to 12.5%, or the II type error is equal to 87.5%.

So, the low power could lead to accepting the null hypothesis of equality between groups even if there was an actual difference in the population. This is due to the low sample size.

**Figure 6:**
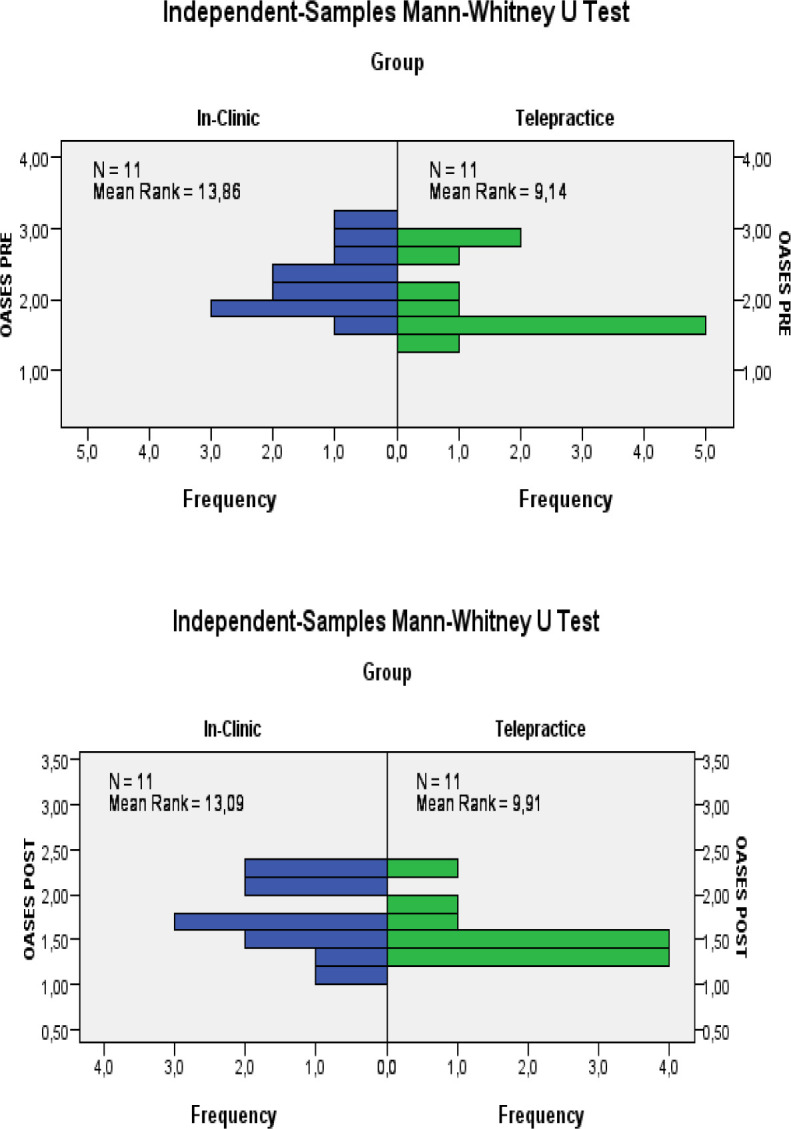
Comparison OASES's Total Score

**Figure 6** compares the results obtained at the pre- and post-treatment assessments of the two groups on the OASES questionnaire.

To statistically compare the results obtained through the two different modes of delivery, a statistical analysis was carried out on the data collected through the application of a two-tailed Student's T test for independent groups and the Mann Whitney test for independent groups. For OASES pre-treatment we obtained t_20_=−1.288; p=0.213 and Mann Whitney U = 86.5; n1 = n2 = 11; p= 0.088 and for OASES post-treatment we obtained t_20_=−1.211; p=0.240 and Mann Whitney U = 78; n1 = n2 = 11; p= 0.270. This data indicates that there is no statistically significant difference between the results obtained in-person and in telepractice on the improvement of covert aspects.

We calculated the power of the two tests also in this case. In the first case the power is equal to 25.1%, or the II type error is equal to 74.9%, while in the second case it is equal to 22.7%, or the II type error is equal to 77.3%.

So, the low power could lead to accepting the null hypothesis of equality between groups even if there was an actual difference in the population. This is due to the low sample size.

**Table 5** compares the missed appointment's rate of the two groups. The In-Clinic Group had missed a mean of 8,15% of the total therapy sessions. The Telepractice Group had missed a mean of 6,66% of the total therapy sessions. We analyzed this data through a two-tailed Student's T test for independent groups and the Mann Whitney test for independent groups. We obtained t_20_=−0.64; p=0.530 and Mann Whitney U =70; n1 = n2 = 11; p= 0.562. This data shows that there is not a significant difference between the number of therapy sessions delivered by telepractice and those delivered in clinic. We calculated the power of the two tests also in this case: it is equal to 9.7%, or the II type error is equal to 90.3%

**Table 5:** Comparison of the In-Clinic Group and Telepractice Group Missed Appointments Rate

IN-CLINIC GROUP	MISSED APPOINTMENT RATE	TELEPRACTICE GROUP	MISSED APPOINTMENT RATE
I1	13,04%	T1	5,10%
I2	8,00%	T2	0,00%
I3	10,71%	T3	5,26%
I4	0,00%	T4	3,39%
I5	11,54%	T5	5,26%
I6	16,07%	T6	3,39%
I7	13,79%	T7	6,84%
I8	10,14%	T8	17,89%
I9	5,13%	T9	8,62%
I10	0,00%	T10	14,00%
I11	1,22%	T11	3,51%

So, the low power could lead to accepting the null hypothesis of equality between groups even if there were differences. This is due to the low sample size.

**Figure 7:**
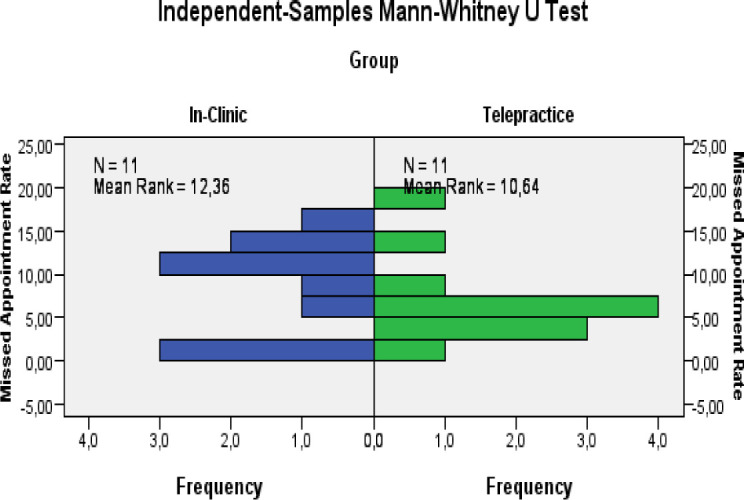
Comparison of Missed Appointment Rate for In-Clinic and Telepractice Group

Thus, to test whether the non-significance of differences persists even in situations with adequate statistical power, we suggest that future studies increase the sample size.

## DISCUSSION

The present study aimed to investigate the efficacy of the MIDA-SP treatment program for developmental stuttering delivered by telepractice, comparing it with the results obtained through the same treatment delivered in an in-person historical control group.

The data obtained through the administration of the SSI-4 test, before and after treatment, both for the In-Clinic Group and the Telepractice Group, showed that both delivery methods led to a decrease in the frequency and average duration of episodes of disfluency and a reduction in the occurrence of physical concomitants. The statistical analysis carried out on the results obtained in the two groups resulted in a non-significant p-value (p >0.05). Therefore, there was no statistically significant difference between the two groups. This means that the treatment of developmental stuttering through the MIDA-SP program delivered by telepractice did not lead to a lowering of quality standards compared to the same treatment delivered in person.

Although there was no change in severity range, the analysis of data collected before and after treatment in both groups through the administration of the OASES questionnaire, showed that in both delivery methods there was a decrease in the total score. These results suggest that there were clinically significant differences.

Qualitative analysis of the results showed an increase in the children's awareness of both the disorder and how it manifests itself in both groups. There was a reduction in the emotional implications of the disorder and an improvement in aspects concerning quality of life, both in the children treated in-person and in the children treated by telepractice. There was also a reduced impact of the disorder on the daily life of the children. The statistical analysis carried out on the results returned a p-value higher than the 0.05 threshold (p = 0.2). Telepractice delivered MIDA-SP treatment of the covert aspects of developmental stuttering can be considered effective and did not lead to a lowering of quality standards compared to the same treatment delivered in person.

The fact that the value of the power is low, suggests that we could accept the null hypothesis of equality between the groups we analyzed, but further analysis is needed to extend this result. Another aim of this study was to analyze the rate of missed appointments between the two groups. It was found that the Telepractice Group had a lower rate of absences, although not statistically significant. In addition, most of the absences in the Telepractice Group occurred during the summer holidays following the restrictions due to the COVID-19 pandemic.

### LIMITATIONS

The present study was conducted during the critical phase of the COVID-19 medical emergency. This situation led to a rapid reorganization of the therapy delivery system. The consequences of this rapid reorganization were (a) the absence of blinding at the time of patient assessment (i.e., assessments were performed by the clinicians who provided the therapy); (b) the replacement of art-mediated training with an enhancement of transfer activities. Another limitation was related to the small sample of patients.

### FUTURE DIRECTIONS

A recommendation for future research is to increase the sample size to verify the generalizability of the results. A larger sample size would provide greater statistical power. Also, the research could be expanded with a mixed delivery method sample. This would allow us to understand whether effectiveness is maintained while changing the mode of delivery. The consequence of this would be to have greater flexibility in the delivery of services, to better meet the needs of patients, and to optimize costs and resources. In addition, the study could be extended to different age groups.

Finally, it would be interesting to replicate the efficacy study of MIDA-SP via telepractice that includes art-mediated training.
